# Synergistic Enhancement of Antioxidant Activity in *Arbutus unedo* Leaf Extract by Biological Fluids: Implications for Functional and Nutraceutical Applications

**DOI:** 10.1002/bab.70164

**Published:** 2026-03-31

**Authors:** Idir Moualek, Hamdi Bendif, Karima Benarab, Giulio Lupidi, Fehmi Boufahja, Stefania Garzoli, Karim Houali

**Affiliations:** ^1^ Laboratory Analytical Biochemistry & Biotechnology Research, Faculty of Biological Sciences and Agricultural Sciences Mouloud Mammeri University of Tizi‐Ouzou Tizi‐Ouzou Algeria; ^2^ Biology Department, College of Science Imam Mohammad Ibn Saud Islamic University (IMSIU) Riyadh Saudi Arabia; ^3^ University Hospital Center of Tizi‐Ouzou Tizi‐Ouzou Algeria; ^4^ School of Pharmaceutical Sciences and Health Products University of Camerino Camerino Italy; ^5^ Department of Chemistry and Technologies of Drug Sapienza University Rome Italy

**Keywords:** *Arbutus unedo*, HPLC‐MS Analysis, biological fluids, functional foods, polyphenols, Antioxidant Power

## Abstract

This study examined the effect of biological fluids (saliva, blood plasma, and urine) on the antioxidant capacity of an aqueous extract derived from *Arbutus unedo* leaves. The extract displayed a concentration‐dependent reducing activity in the ferric reducing power assay (IC_50_ = 292 ± 7.54 µg/mL) and phosphomolybdenum assays (IC_50_ = 461.67 ± 4.16 µg/mL), indicating considerable antioxidant potential, albeit inferior to ascorbic acid (IC_50_ = 56.72 ± 2.79 and 156.55 ± 7.40 µg/mL, respectively). The investigation via high‐performance liquid chromatography‐mass spectrometry (HPLC‐MS) discovered significant phenolic substances, such as quinic acid, arbutin, epicatechin, and quercetin derivatives, which likely contribute to the reported effects. The interaction with biological fluids enhanced the antioxidant activity synergistically. Saliva (50 µL) enhanced molybdenum reduction by 10%, but plasma (25 µL) augmented it by 27.8% relative to the extract alone. Urine (25 µL) exhibited the most significant effect, increasing molybdenum reduction by 30.24%. Likewise, iron reduction was enhanced by 32.00% with plasma and 19.00% with urine. The observed effects were ascribed to interactions between polyphenols and proteins or endogenous antioxidants, such as uric acid, in bodily fluids. The results underscore the significance of physiological context in assessing plant‐based antioxidants, indicating that *A. unedo* extract may exhibit increased *bioactivity* in vivo due to synergistic interactions with biological components. The study supports the potential of *A. unedo* as a functional food ingredient and highlights the importance of considering biological fluid interactions during nutraceutical formulation.

## 1 Introduction

Polyphenols, abundant in plant‐derived foods, are recognized for their antioxidant and anti‐inflammatory properties [1]. However, their bioavailability and biological activity are significantly influenced by interactions with proteins present in biological fluids such as saliva, blood, and urine [1, 2].

Salivary proteins, in particular, play a crucial role. Proline‐rich proteins and histatins are effective at binding polyphenols like tannins, which can modulate taste perception and provide a first line of defense [3]. This interaction directly influences the perceived astringency and acceptability of polyphenol‐rich foods [4].

In the bloodstream, polyphenols interact with plasma proteins, notably serum albumin. This binding affects their distribution, metabolism, and ultimate biological activity [5], potentially altering their therapeutic potential in various pathologies. The study of such interactions is a cornerstone of ethnopharmacology and integrative medicine, helping to explain and validate the physiological effects of traditional remedies [6].

*Arbutus unedo* L., commonly known as strawberry tree, is a prime example of a Mediterranean plant with significant ethnopharmacological value, historically used for its diverse therapeutic properties [7]. Modern phytochemical research is essential to validate such traditional uses. Advanced techniques like LC/MS/MS are pivotal for characterizing the complex phenolic profiles of natural extracts, from bee products to medicinal plants, and for identifying key bioactive constituents such as flavonoids and phenolic acids [8]. Applied to *A. unedo*, these methods confirm its richness in diverse phenolic compounds, including phenolic acids, flavonoids, and tannins, which vary with factors like season and origin [9].

This study aims to elucidate lies the elucidation of the symbiotic relationship between biological fluids and the physiological status quo as a model to understand how this interaction affects the antioxidant and anti‐inflammatory potential of *A. unedo* polyphenols. The results contribute to the current knowledge on the antioxidant properties of *A. unedo* and provide a foundation for further exploration of its therapeutic potential of plant extracts in combating oxidative stress‐related disorders.

## 2 Material and Methods

### 2.1 Plant Material and Extract Preparation

Leaves were collected from *A. unedo* plants in the Tizi‐Ouzou region of Algeria during the month of December 2023. The plant specimens were properly identified and authenticated by Professor Mahmoud Laribi, an expert botanist affiliated with the Department of Plant Biology at Mouloud Mammeri University of Tizi‐Ouzou. A voucher sample of the identified plant material was deposited and preserved at the university's facilities.

The harvested leaf samples were air‐dried and subsequently ground into a fine powder form. This powdered material was stored away from light exposure at room temperature until the extraction process began. To prepare the extract, 20 g of powder was mixed with 200 mL of distilled water and allowed to macerate for a 24‐h period at room temperature. Following this maceration step, the liquid portion was isolated by filtration and then underwent lyophilization to obtain the final dried extract.

### 2.2 Blood Sampling

Venous blood samples were collected from healthy, nonsmoking volunteers who were not on any medication. The samples were collected in heparinized tubes and stored at 4°C.

### 2.3 Saliva Sampling

Saliva samples were collected at rest using sterile cannulas from healthy, nonsmoking volunteers who did not chew tobacco and had no lesions or pathologies. The samples were collected in sterile Falcon tubes and stored at 4°C.

### 2.4 Urine Sampling

Urine samples were collected from healthy volunteers according to the guidelines for conducting a urine cytobacteriological examination (UCBE). This involved thoroughly cleaning the urinary meatus with disinfectant wipes, discarding the initial stream, and then collecting the sample in an UCBE container, which was stored at 4°C.

### 2.5 Ferric Reducing Power Assay

Reducing power was determined by the method described by Hazra et al. [10] and Oyaizu [11]. Different concentrations of extract were mixed with 1.25 mL of 0.2 M, pH 6.6 sodium phosphate buffer and 1.25 mL of potassium ferricyanide (1%). The mixture was incubated at 50°C for 20 min. After incubation, the reaction mixture was acidified with 1.25 mL of trichloroacetic acid (10%) and centrifuged at 3000 rpm for 10 min. Finally, 0.5 mL of freshly prepared FeCl_3_ (0.1%) was added to this solution, and the absorbance was measured at 700 nm. Ascorbic acid at various concentrations was used as the standard.

### 2.6 Total Antioxidant Capacity

Total antioxidant capacity was estimated by phosphomolybdenum assay [12, 13]. The tubes containing extract and reagent solution (0.6 M sulfuric acid, 28 mM sodium phosphate, and 4 mM ammonium molybdate) were incubated at 95°C for 90 min. Then, the solution was cooled to room temperature, and absorbance was read at 695 nm.

### 2.7 Plant–Biological Fluids Interaction

The pharmacological application of the aqueous extract of *A. unedo* leaves involves determining pharmacokinetic parameters that provide insights into the interactions between bioactive molecules and those within the organism during their journey. In this context, we studied the effect of saliva, blood, and urine on the antioxidant effect of the extract.

This aspect of our study was inspired by the work of Bennick [3] on the interaction of polyphenols with salivary proteins. Building on this foundation, we focused on the interaction of the *A. unedo* extract with different biological fluids (saliva, blood, and urine).

### 2.8 Effect on Molybdenum Reduction

The technique used is described by Prieto et al. [12].

First, we optimized the volume of each biological substance to induce a maximal reducing capacity as follows:

‐ For saliva, this determination is performed using four volumes (25, 50, 75, and 100 µL).
‐ For blood, plasma is separated from red blood cells by centrifuging 3 mL of blood at 300 *g* for 10 min at 4°C. Then, four volumes of plasma (25, 50, 75, and 100 µL) are tested.
‐ For urine, five volumes are tested (5, 10, 15, 20, and 25 µL).

### 2.9 Effect on Iron Reduction

The method used is described by Oyaizu [11].

First, we optimized the volume or concentration of each biological substance studied as follows:

‐ For saliva, the volume inducing significant ferric ion reducing power is determined using eight volumes (ranging from 50 to 400 µL).
‐ For blood, plasma is separated from red blood cells by centrifuging 3 mL of blood at 300 *g* for 10 min at 4°C. Then, five volumes are tested (50, 100, 200, 300, and 400 µL).
‐ For urine, 13 volumes are tested (ranging from 1 to 45 µL).

### 2.10 Use of Ascorbic Acid as Standard

In both assays, we established a threshold value using ascorbic acid as our reference standard. This threshold corresponds to the absorbance at which the ascorbic acid curve begins to plateau, in our experimental conditions, and we designated this value as our 100% reduction reference point.  For all subsequent measurements (extract alone, biological fluids alone, and combinations), we calculated the percentage of reduction relative to this ascorbic acid threshold. This approach allowed us to express our results as a percentage of the maximum reducing capacity observed with respect to our reference standard, even though we are measuring an increase in absorbance in the assays.

### 2.11 Calculation of Synergism Between Antioxidants

The synergistic effects of a combination of different samples (saliva, plasma, and urine) with aqueous extract of *A. unedo* on phosphomolybdenum assay and ferric ion reducing assay were calculated as the ratio between the obtained inhibition (I) and the calculated expected inhibition (IE) [14]

The IE was calculated as follows:

$$\left(A+B\right)-\left(A\times B/100\right)=\mathrm{Expected}\mathrm{inhibition}\left(\mathrm{IE}\right)$$

where *A* is inhibition by antioxidant alone (% of antioxidant activity with no added plant extract) and *B* is inhibition by plant extract alone at the different concentrations used in the combination assays (% of control). Synergism is proven if the ratio *I*/IE is greater than 1.

### 2.12 High‐Performance Liquid Chromatography MS TOF Analysis

Phenolic characterization was carried out using an Agilent 1200 Liquid Chromatography system (Agilent Technologies, Palo Alto, CA, USA) equipped with a standard autosampler. The HPLC‐MS column was an Agilent Extended C18 (1.8 µm, 2.1 × 50 mm).

Separation was carried out at 40°C with a gradient elution program at a flow rate of 0.5 mL/min. The mobile phases consisted of water plus 0.1% formic acid (A) and acetonitrile (B). The following multistep linear gradient was applied: 0 min, 1% B; 13 min, 25% B; 19 min, 40% B; 21 min, 90% B. The initial conditions were maintained for 5 min. The injection volume in the HPLC system was 5 µL. The HPLC system was coupled to an Agilent 6320 TOF mass spectrometer (Agilent Technologies, Palo Alto, CA, USA) equipped with a dual ESI interface (Agilent Technologies, Palo Alto, CA, USA) operating in negative ion mode using a capillary voltage of 3.5 kV. The other optimum values of the ESI–TOF–MS parameters were drying gas temperature, 300 C; drying gas flow, 12 L/min; and nebulizing gas pressure, 40 psig. Detection was carried out within a mass range of 50–1700 *m/z*. The accurate mass data of the molecular ions were processed through the software Mass Hunter (Agilent Technologies, Palo Alto, CA, USA).

## 3 Results and Discussion

### 3.1 Total Antioxidant Capacity of the *A. unedo* aqueous extracts

Total antioxidant activity and reducing capacity of the extract were measured using two assays as the phosphomolybdenum and iron reduction assays, and the results are reported in Table 1. The extract exhibited dose‐dependent molybdenum‐reducing activity. Under our experimental conditions, its antioxidant capacity was high, although less potent than ascorbic acid. The findings also revealed that the iron‐reducing capacity of the extract was concentration‐dependent, with a value of IC_50_ that was only three times less potent in comparison to ascorbic acid, which was employed as a positive control.

**TABLE 1:** Total antioxidant activity.

	Molybdenum reduction IC_50_ (mg/mL)	Reduction power IC_50_ (mg/mL)
*Arbutus unedo* extract	461.67 ± 4.16	292 ± 7.54
Ascorbic acid	156.55 ± 7.40	56.72 ± 2.79

### 3.2 Effect of Biological Fluid Interaction on the Reduction of Molybdenum and Iron

Figure 1A–C reports the results obtained for the molybdenum reduction activity of samples of saliva, blood plasma, and urine. All biological samples showed reducing activity in a dose‐dependent manner at all concentrations tested, and in particular, the quantities of saliva, blood plasma, and urine that showed the highest reducing capacity were 55.12 ± 0.53% for 50 µL of saliva, 70.33 ± 0.6% for 25 µL of plasma, and 97.05 ± 0.35% for 25 µL of urine.

**FIGURE 1:**
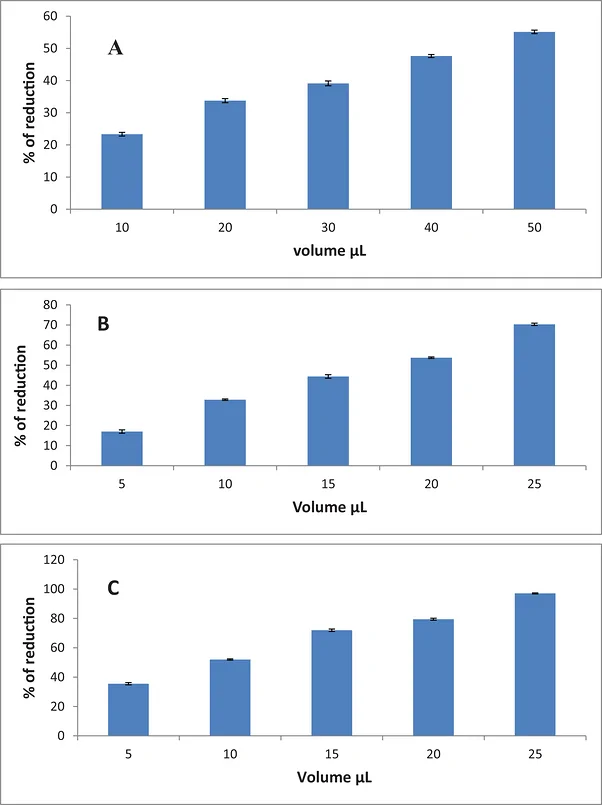
Percentage of molybdenum reduction as a function of (A) saliva volume, (B) plasma volume, and (C) urine volume.

Regarding iron reduction, from data of Figure 2A–C, the quantities of saliva, blood plasma, and urine that showed the higher iron reduction power were, respectively, 400 µL (28.31 ±0.79%) for saliva, 400 µL (78.12 ± 0.62%) for blood plasma, and 20 µL (98.81 ± 0.82%) for urine.

**FIGURE 2:**
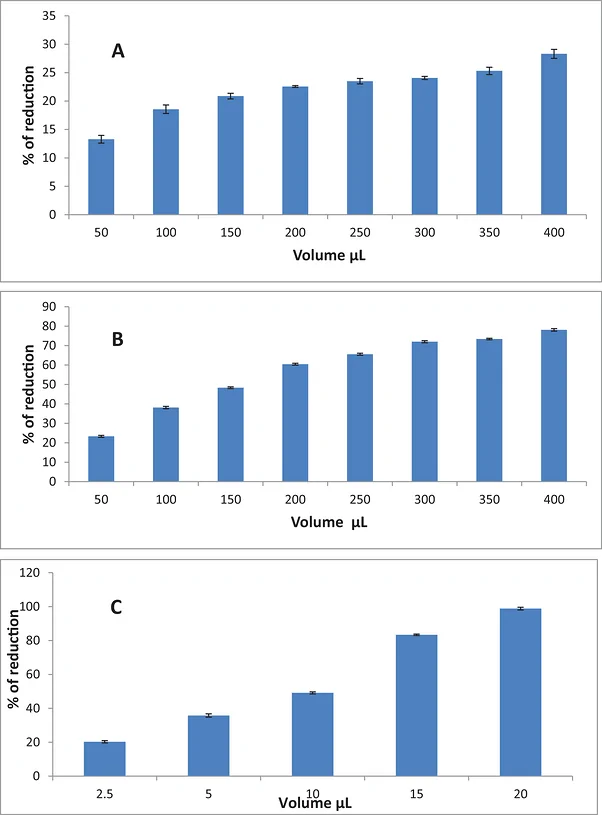
Percentage of iron reduction of (A) saliva volume, (B) blood plasma volume, and (C) urine volume.

### 3.3 Effect of Plant Extract of *A. unedo*–Biological Fluid Interaction on the Reduction of Molybdenum and Iron

The antioxidant capacity of the combination of the extract with each biological fluid (saliva, blood plasma, and urine) on the reduction of molybdenum and iron by the extract was evaluated by combining an optimal predetermined quantity of the biological compound with different extract concentrations. As indicated in Figures 3, 4, 5, we observed that this combination gives the mixture a higher molybdenum‐reducing activity than that recorded with each compound alone. All combinations describe a synergy resulting in an activity superior to that of the extract and the standard.

**FIGURE 3:**
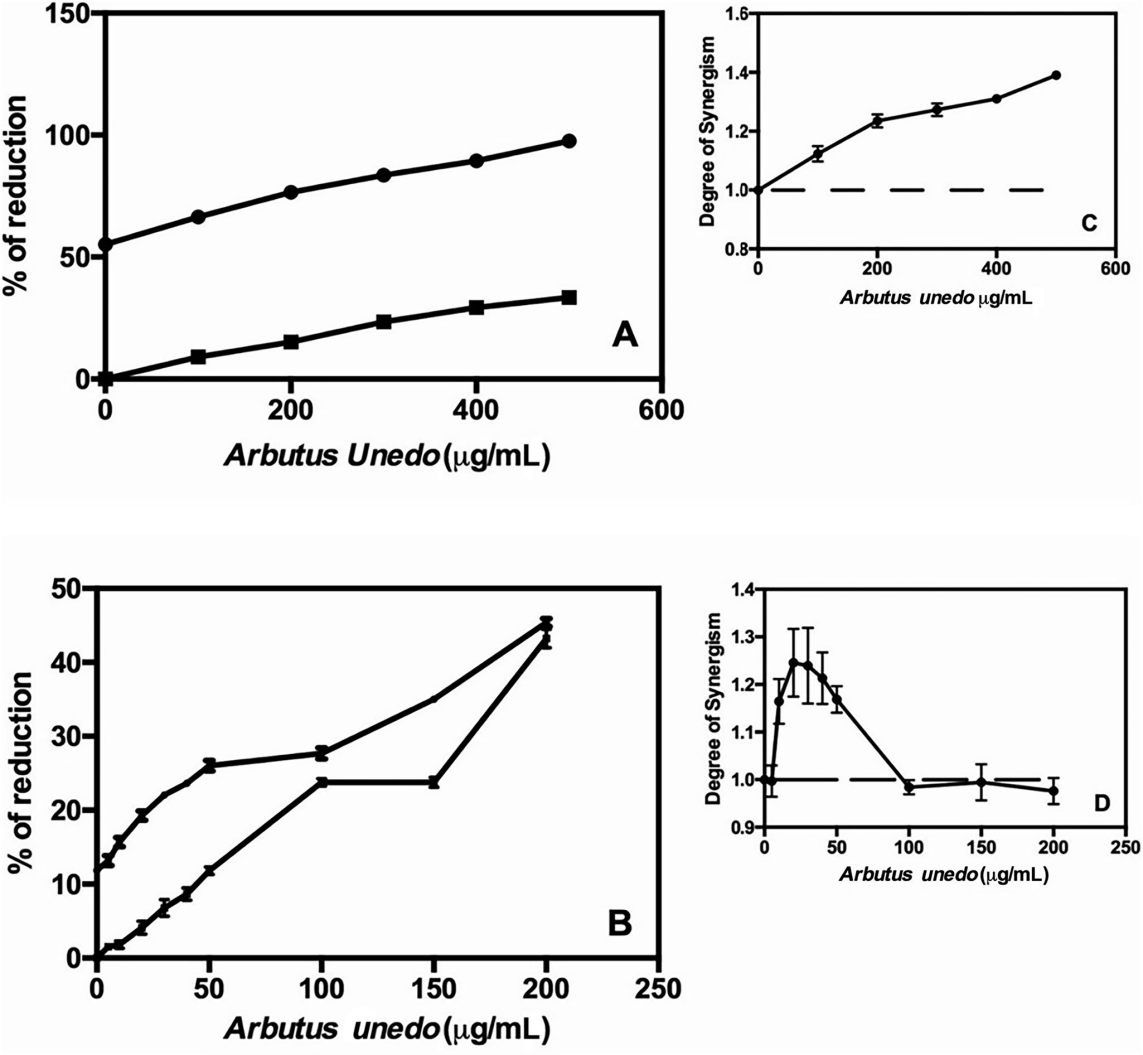
Effects of the combination with extract of *Arbutus unedo* with saliva (50 µL) (•) on molybdenum reduction (A) and on iron reduction (B) compared to the extract alone (■). (C) and (D) calculated the degree of synergism between saliva and *A. unedo* concentration

**FIGURE 4:**
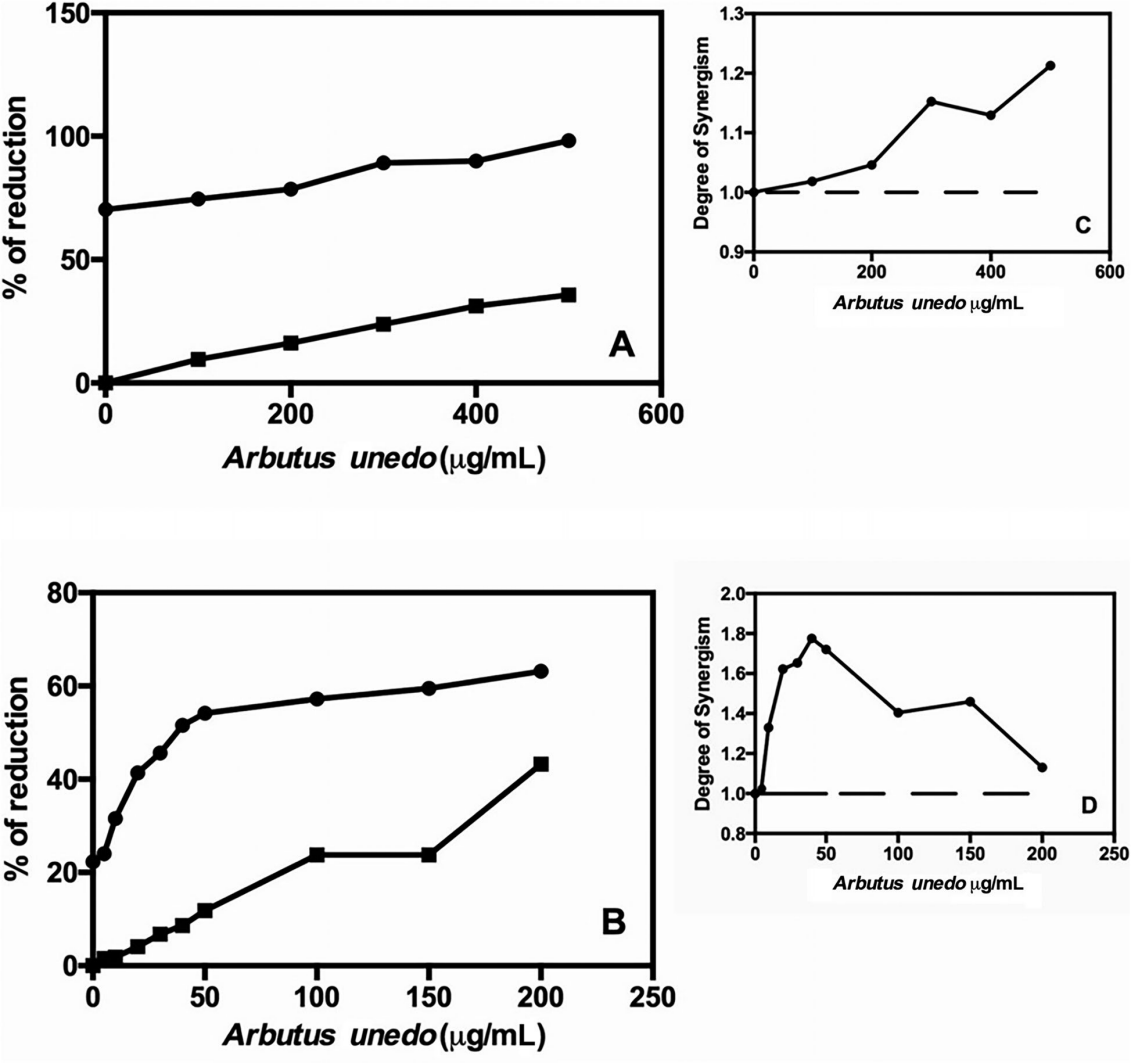
Effects of the combination with extract of *Arbutus unedo* with human plasma (—— µL) (•) on molybdenum reduction (A) and on iron reduction (B), compared to the extract alone (■). (C) and (D) calculated the degree of synergism between plasma and *A. unedo* concentration

**FIGURE 5:**
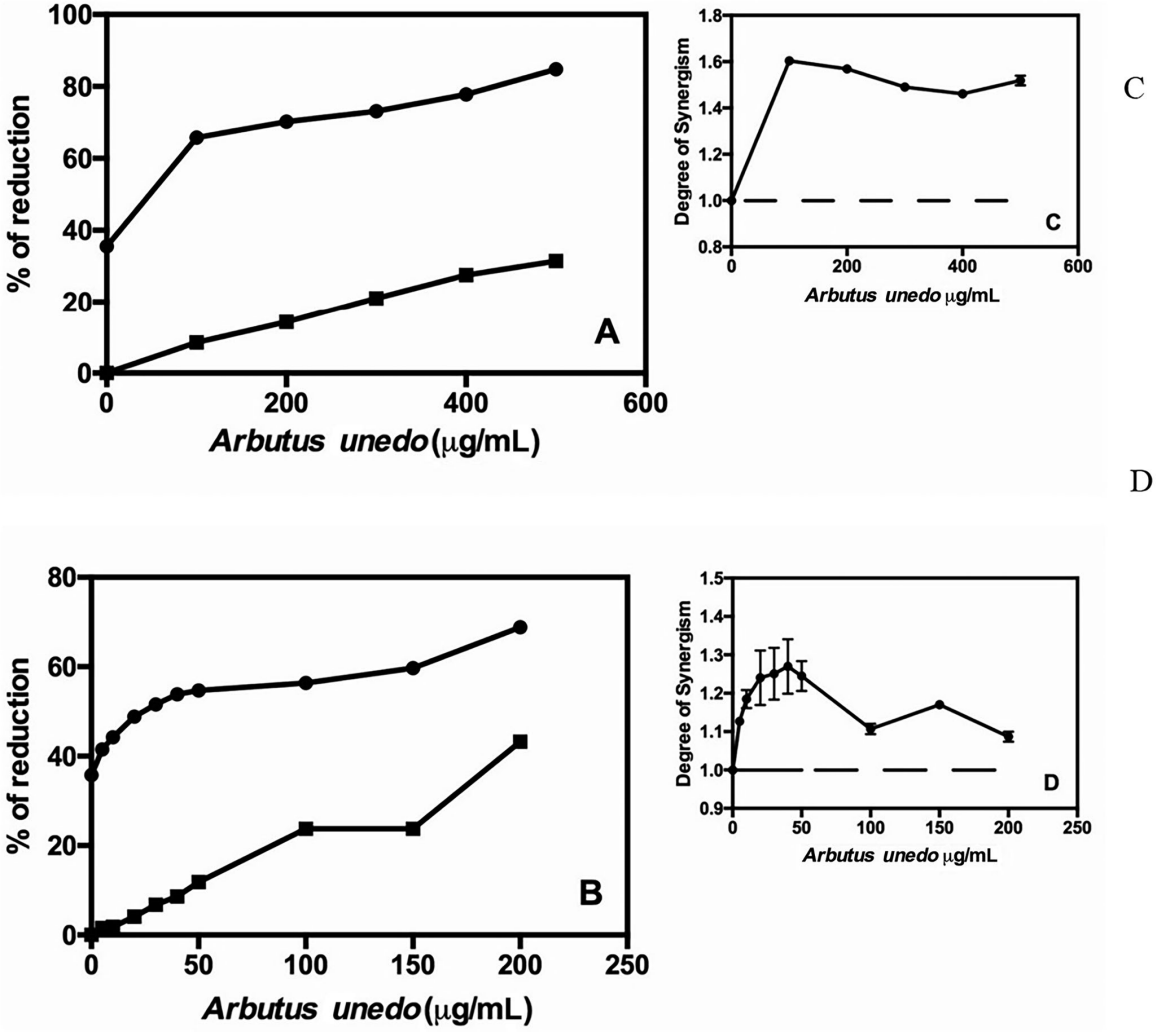
Percentage of molybdenum reduction (A) and percentage of iron reduction (B) by urine sample (•) (5 µL in the assay) incubated with different concentrations of *Arbutus unedo* extract, compared to the extract alone (■). (C) and (D) calculated the degree of synergism between urine and *A. unedo* concentration.

Furthermore, from the same figures, we can clearly observe that the combination with plant extract gives the mixture a higher iron‐reducing power than that recorded individually with each compound. These results indicate that all combinations describe the existence of a synergy resulting in an activity greater than that recorded for the extract. Nevertheless, compared to the standard, all combinations display a lower maximum activity.

### 3.4 Plant–Saliva Combination

As reported in Figure 3A, the combination of extract with saliva results in a positive increase of maximum molybdenum‐reducing activity of about 10% compared to the extract alone for all concentrations used, suggesting a synergic effect between saliva and *A. unedo*. The calculated degree of synergism between saliva and plant extract on the assay was dose‐dependent (Figure 3C)

The effects of the combination of the extract of *A. unedo* with saliva on iron‐reducing power were reported in Figure 3B. The results indicate that all combinations of saliva with the extract display a lower effect on the total iron‐reducing effect.

This combination gives a maximum reducing activity of 45.37 ± 0.58 (saliva plus 200 µg of *A. unedo* extract) with an increase of only 2.15% compared to the extract alone (43.22%). The calculated degree of synergism between saliva and plant extract in the iron reduction seems dose‐dependent (Figure 3D), and it reaches its maximum values at a low rate between saliva and plant, with a rapid decrease as the concentration of *A. unedo* extract increases.

Salivary proteins serve as a screening mechanism that allows the absorption of flavonoids and the exploitation of their beneficial effects, but neutralize the less desirable effects of tannins. It is generally accepted that tannins are synthesized by plants to act as deterrents for animals due to their bitter and astringent properties.

These can interfere with the growth and health of livestock and could potentially be harmful to humans [3]. The high affinity of polyphenols for oral surfaces contributes to maintaining their slow release and thus helps to create a higher redox state that could help better cope with oxidative stress.

The ability of salivary antioxidants to act synergistically with natural antioxidants such as polyphenols influences the maintenance of a high antioxidant potential in the oral cavity and thus confers increased resistance to infections [15]. The variation in this capacity is attributed to several factors and indicates either an exogenous antioxidant supply or the degree of depletion of these antioxidants. Thus, low values of molybdenum reduction are recorded in patients with periodontitis and are negatively correlated with the severity of the condition [16]. Furthermore, these low values can be explained by the fact that the immune response against periodontopathogenic germs is associated with increased ROS production by neutrophils and macrophages. To avoid destruction of host tissues, these ROS are neutralized by antioxidants, which could lead to a decrease in the reducing power of molybdenum and iron [17].

The increase in salivary reducing potential can be attributed to diet. The sum of endogenous and food‐derived antioxidants represents the total antioxidant activity of the system. Uric acid is the main antioxidant present in human saliva, accounting for more than 85% of the reducing power of saliva and mainly derived from the diet [18]. On the other hand, this increase is explained by the fact that antioxidant levels could be modified in response to an infection or disease.

### 3.5 Plant–Blood Plasma Combination

In blood plasma, the combination with the extract increased molybdenum‐reducing activity (Figure 4A) of plasma equal to 98.13 ± 0.53% (when incubated with 500 µg of extract), with an increase of 27.8% compared to the activity of the plasma alone, while the extract at the same concentration showed a molybdenum‐reducing activity equal to 35.66%. The degree of synergism calculated between plasma and the plant extract demonstrated a low dose‐dependent behavior (Figure 4C), with a minimal increase in the plasma's reducing activity as a function of the concentration of *A. unedo*.

More interesting is the effect of the association of extract with plasma on the reducing power reported in Figure 4B where we can observe a progressive increase of reduction power of the association plasma–extract when we incubated with concentration of extract from 0 to 50 µg. In fact, we observed an apparent synergic effect with an increase of the 32.00% (incubation with 50 µg of extract) with respect to the activity of the extract alone at the same concentration. An increase in the concentration of extract above 50 µg affects the iron‐reducing power, decreasing proportionally to reach a minimum value at maximum concentration used (200 µg, 35.66% of activity), probably due to a saturation effect produced by the large concentration of extract used compared to the µL of plasma used in the assay. The calculated degree of synergism between plasma and plant extract agreed with the observed behavior (Figure 4D).

According to Devaraj et al. [19], supplementation with polyphenols extracted from *Pinus pinaster* increases the antioxidant capacity of blood plasma by 40%. Similar results are reported by Pulido et al. [20] in the case of ingestion of polyphenol‐rich beverages such as red wine, fruit and vegetable juices, tea, or coffee.

Another study conducted by Teissedre [21] highlights the physiological effects obtained by wine consumption such as a restoration of the antioxidant capacity of the plasma, which allows an improvement of the defenses against oxidative stress in diabetes, as well as a favorable action on insulin secretion. On the other hand, Pulido et al. [20] evaluated the effect of other polyphenol‐rich beverages and found that the ingestion of these beverages, such as red wine, fruit and vegetable juices, or coffee and tea, is likely to favorably impact the antioxidant potential of the plasma.

Regarding tea, renowned for its richness in flavonoids, the study conducted by Leenen et al. [22] shows that its ingestion leads to a significant increase in the antioxidant status of the plasma, with a significantly greater effect for green tea compared to black tea.

Polyphenols are known for their interactions with plasma proteins in the blood, which are formed by hydrophobic interactions. The influence of this interaction on the plasma antioxidant potential can only be determined by an antioxidant analysis and by taking into account the structural characteristics of the polyphenols as well as the proteins [23]. In the blood, polyphenols have a strong affinity for plasma proteins, which depends on several factors such as the number of hydroxyl groups, the presence or absence of a double bond, and the glycosylation of the polyphenols. In the case of flavonoids, glycosylation decreases their affinity for plasma proteins, while their methylation increases it. The interaction between polyphenols and proteins in the blood is a complex phenomenon that is not yet elucidated.

In addition, the competitive effect for these proteins exerted by numerous blood molecules, such as metal ions, glucose, fatty acids, and numerous metabolites, can interfere with polyphenols. Recently, it has been found, in vitro, that metal ions such as Al^3+^, Zn^2+^, Cu^2+^, and Fe^3+^ significantly reduce the interaction of several flavonoids with albumin [24–26].

In the blood, the antioxidant capacity is strongly affected by uric acid concentrations, which, in addition to being a potent free radical scavenger, provide from 60% to 80% of the antioxidant potential in the plasma. Lin et al. [27], report that, in vitro, quercetin showed an inhibitory effect on xanthine oxidoreductase, an enzyme involved in the final step of intracellular uric acid production.

These data suggest that quercetin could lower blood uric acid concentrations and thus decrease the antioxidant capacity of the blood.

The physiological effects obtained from the consumption of polyphenols induce a restoration of the antioxidant capacity of the plasma, an improvement in defenses against oxidative stress in diabetes, a reduction in plasma glucose concentrations, an increase in insulin secretion, and an increase in tissue sensitivity to insulin [21, 28–30].

There is a positive correlation between polyphenol concentration and plasma antioxidant potential. Nevertheless, the plasma polyphenol concentrations reached after their consumption vary greatly depending on their nature and dietary source [31]. According to Duthie et al. [29] and Young et al. [30], the rate of plasma absorption of polyphenols depends on their chemical structure and not on their concentration. The polyphenols best absorbed in humans are isoflavones, gallic acid, followed by catechins, flavanones, and quercetin glucosides, while the least well absorbed are proanthocyanidins, galloylated tea catechins, and anthocyanins.

The beneficial effects of these polyphenols depend on various factors, such as total intake, food cooking processes, digestion, absorption, metabolic pathways, and also individual differences.

### 3.6 Plant–Urine Combination

In the case of urine, the effect of the combination with the extract on molybdenum‐reducing activity (Figure 5A) shows a high synergic effect (Figure 5C) on the reduction activity of 30.24% in the presence of 100 µg of extract with respect to the urine alone. When incubated with a higher concentration of extract, the activity of the combination of urine–extract remains constant (49.24% respect to plasma alone) even at the maximum concentration of extract used (500 µg of extract, 31.47% of activity), probably due to a saturation effect given the small quantity of urine used in the test.

Also, in the case of iron‐reducing activity, the combination of urine with extract (Figure 5B) from 0 to 50 µg, shows a positive synergic effect (Figure 5D) on iron reduction activity, with an increase of about 19.00% respect to the activity of extract alone at the same concentration (50 µg of extract). Incubation of the urine sample with a higher concentration of the extract shows a small increase in the observed total iron‐reducing activity, reaching 68.81% compared to 43.22% shown by the extract alone at the same concentration used in the combination experiment (500 µg).

The composition of urine is unpredictable, reflecting the constantly evolving environment of the body, which is influenced, among other things, by diet.

Approximately 160 polyphenols have been described in urine; their quantity is positively correlated with plasma concentrations, and their measurement is used as a biomarker of dietary intake [32, Hanhineva 2010, 33]. Hippuric acid, a urinary indicator of polyphenol consumption [34], is a metabolite resulting from the microbial degradation of polyphenols in the colon [35]. It has also been reported that the reducing potential of urine is mainly linked to the uric acid concentration of the samples. The latter represents approximately 75% of the antioxidant potential of urine

The biological potential of urinary polyphenols depends on their specificity, sensitivity, variability, intake, and bioavailability [32, Hanhineva 2010, 33]. In the urinary system, a regional selectivity of polyphenols in the prostate has been reported. This characteristic, combined with the fact that polyphenols such as those from green tea have a protective effect against prostate cancer, underscores the importance of polyphenols in the urinary tract [36, 37].

For example, anacardic acid is capable of inhibiting the angiogenesis of prostate tumors by targeting the proto‐oncogene signaling pathway. Moreover, it affects the viability of endothelial cells, migration, adhesion, and differentiation both in vitro and in vivo of cancer cells [38]. While resveratrol is involved in inhibiting the growth of cancer cells, inducing cell cycle arrest and apoptosis in various prostate cancer cell lines [39, 40], it has been shown that dietary polyphenols influence the risk of developing prostate cancer by regulating inflammatory genes and reducing oxidative DNA damage [41, 42].

### 3.7 High‐Performance Liquid Chromatography MS TOF Analysis

The HPLC analysis identified several phenolic compounds in the aqueous extract of *A. unedo* leaves (Table 2, Figure 6), including quinic acid, arbutin, galloylquinic acid, galloylshikimic acid, gallic acid methyl ester, epicatechin, digalloyl shikimic acid, quercetin derivatives (quercetin 3‐O‐glucoside and quercetin 3‐O‐xyloside), ellagic acid rhamnoside, and kaempferol derivatives (kaempferol xyloside and kaempferol rhamnoside). These compounds are known for their antioxidant properties and could contribute to the observed reducing effects on iron and molybdenum.

**TABLE 2:** Molecules identified by HPLC in *Arbutus unedo* aqueous extracts.

Peak	Compound	Rt (min)	Molecular formula proposed (M‐H)^−^	*m/z* experimental	*m/z* calculated	Error (ppm)
1	Quinic acid	0.456	C_7_H_12_O_6_	191.0533	191.0561	14.87
2	Arbutin	0.738	C_12_H_15_O_7_	271.0876	271.0895	3.07
3	Galloylquinic acid	1.050	C_14_H_15_O_10_	343.0746	343.0756	1.10
4	Unknown	1.810	ND	305.0727	305.0725	−0.08
5	Galloylshikimic acid	2.431	C_14_H_13_O_9_	325.0637	325.0637	2.37
6	Gallic acid methyl ester	4.350	C_8_H_7_O_5_	183.0326	183.0299	−14.74
7	Unknown	4.450	ND	345.0896	ND	ND
8	Epicatechin	5.411	C_15_H_13_O_6_	289.0774	289.0776	1.14
9	Unknown	6.105	C_12_H_24_O_16_	423.1024	423.0992	−7.34
10	Digalloyl shikimic acid	6.850	C_21_H_17_O_13_	477.0774	477.0733	−8.06
11	Unknown	7.550	ND	431.2010	431.2008	−0.38
12	Quercetine galloyl derivate	8.950	ND	615.1117	ND	ND
13	Quercetin 3‐O‐glucoside	9.250	C_21_H_19_O_12_	463.0975	463.0975	−3.5
14	Quercetin 3‐O‐xyloside	10.370	C_20_H_17_O_11_	433.0864	433.0862	−0.2
15	Ellagic acid rhamnoside	10.630	C_20_H_15_O_12_	447.1023	447.0992	−3.1
16	Kaempferol xyloside	11.380	C_20_H_17_O_10_	417.0909	417.0886	−2.3
17	Kaempferol rhamnoside	11.705	C_21_H_19_O_10_	431.1067	ND	−5.43

Abbreviation: ND, not determined.

**FIGURE 6:**
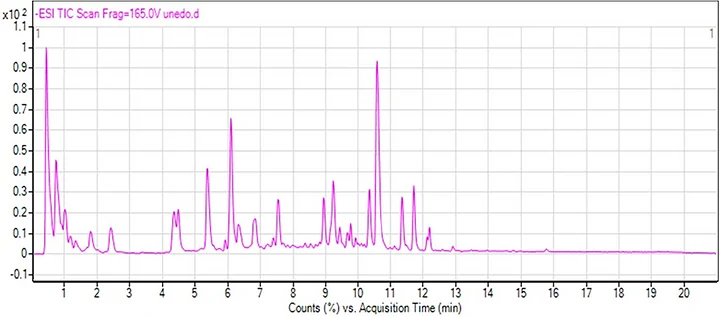
Chromatogram of molecules identified by HPLC in *Arbutus unedo* aqueous extracts.

Quinic acid and its derivatives, such as galloylquinic acid and galloylshikimic acid, have been reported to possess antioxidant and metal‐chelating properties [43, 44]. These compounds could potentially chelate iron and molybdenum ions, thereby influencing their reduction.

Arbutin, a glycosylated hydroquinone derivative, has been shown to exhibit antioxidant activity and can act as a reducing agent [45]. Its presence in the extract could contribute to the observed reducing effects.

Gallic acid and its derivatives, such as gallic acid methyl ester and digalloyl shikimic acid, are known for their strong antioxidant and metal‐chelating properties [46]. These compounds could play a role in the reduction of iron and molybdenum in the extract.

Epicatechin, a flavan‐3‐ol, has been widely studied for its antioxidant and reducing properties [47, 48]. Its presence in the extract could contribute to the observed reducing effects.

Quercetin and its derivatives, such as quercetin 3‐O‐glucoside and quercetin 3‐O‐xyloside, are well‐known for their potent antioxidant and metal‐chelating activities [49]. These compounds could play a significant role in the reduction of iron and molybdenum by the extract.

Ellagic acid and its derivatives, like ellagic acid rhamnoside, have been reported to possess antioxidant and metal‐chelating properties [50]. Their presence in the extract could contribute to the observed reducing effects.

Kaempferol and its derivatives, such as kaempferol xyloside and kaempferol rhamnoside, are flavonoids known for their antioxidant and metal‐chelating activities [51]. These compounds could also contribute to the reduction of iron and molybdenum by the extract.

Furthermore, the interactions between the extract's phenolic compounds and the constituents of the biological fluids (saliva, blood plasma, and urine) could influence the reducing potential. For instance, the binding of polyphenols to salivary proteins or plasma proteins could affect their availability and reactivity [52, 53]. Additionally, the presence of metal ions, uric acid, and other antioxidants in biological fluids could compete or synergize with the extract's compounds, impacting the overall reducing capacity [54, 55].

## 4 Conclusion

This study presents solid evidence that biological fluids markedly augment the antioxidant potential of *A. unedo* leaf extract via synergistic interactions. The aqueous extract, abundant in bioactive phenolic components including quinic acid derivatives, arbutin, and flavonoid glycosides, exhibited significant ferric ion and molybdenum reduction capacity. The mixing of the extract with human saliva, plasma, and urine resulted in a 30.24% enhancement in antioxidant activity compared to the extract alone, highlighting the crucial influence of physiological matrices on the bioactivity of plant‐derived chemicals. These findings are significant for the development of *A. unedo*‐derived functional foods and nutraceuticals. The observed synergism indicates that the in vivo antioxidant capacity of the extract may surpass estimates from in vitro testing, notably owing to increased activity in plasma, suggesting possible bioavailability advantages when integrated into the human diet. Future research should clarify the molecular interactions between *A. unedo* polyphenols and biological fluids, evaluating the in vivo bioavailability and metabolism of essential compounds, and developing food matrices that enhance these synergistic effects for better nutritional results. This study emphasizes the significance of examining biological fluid interactions in assessing the bioactivity of plant foods and establishes a basis for employing *A. unedo* leaves as a sustainable source of dietary antioxidants. The findings identify this Mediterranean species as a viable candidate for the creation of novel functional food products designed to enhance human health via specific nutritional approaches.

## Author Contributions

Conceptualization: I.M., K.H., and H.B. Methodology: K.B. and G.L. Validation: I.M. and H.B. Formal analysis: F.B. and G.L. Investigation. I.M. and K.B. Resources: G.L. Writing – original draft: I.M. and K.B. Writing – review and editing: K.H., H.B., and S.G. Supervision: K.H., H.B., and S.G. All authors have read and agreed to the published version of the manuscript.

## Ethics Statement

The authors have nothing to report.

[Correction added on May 6, 2026, after first online publication: Funding information section has been removed].

## Conflicts of Interest

The authors declare no conflicts of interest.

## Data Availability

The data will be available upon request to the authors.
